# Comparison of Functional Outcome after Extended versus Super-Extended Pelvic Lymph Node Dissection during Radical Prostatectomy in High-Risk Localized Prostate Cancer

**DOI:** 10.3389/fonc.2017.00280

**Published:** 2017-11-22

**Authors:** Heikki Seikkula, Pieter Janssen, Manuela Tutolo, Lorenzo Tosco, Antonino Battaglia, Lisa Moris, Thomas Van den Broeck, Maarten Albersen, Gert De Meerleer, Hendrik Van Poppel, Wouter Everaerts, Steven Joniau

**Affiliations:** ^1^Department of Urology, Central Finland Hospital District, Jyväskylä, Finland; ^2^Department of Urology, University Hospital, Turku, Finland; ^3^Department of Urology, University Hospitals Leuven, Leuven, Belgium

**Keywords:** urinary incontinence, erectile function, lymph node dissection, radical prostatectomy, prostate cancer

## Abstract

**Background:**

Urinary continence and erectile function (EF) are best preserved when meticulous dissection of prostate and nerve sparing technique are used during radical prostatectomy (RP). However, extent of lymph node dissection (LND) may also adversely affect functional results.

**Objective:**

To determine whether performing a super-extended LND (seLND) has a significant effect on recovery of urinary continence and EF after RP.

**Design, setting, and participants:**

All patients who underwent RP from January 2007 until December 2013 were handed questionnaires assessing continence and EF. All patients in whom at least an extended LND (eLND) was performed were selected. This search yielded 526 patients. 172 of these patients had filed out 2 or more questionnaires and were included in our analysis.

**Outcome measurements and statistical analysis:**

All questionnaires were reviewed. We used Kaplan–Meier analyses and multivariate Cox analysis to assess the difference in recovery of continence and EF over time for eLND/seLND. Primary endpoints were full recovery of continence (no loss of urine) and full recovery of EF (successful intercourse possible). Patients who did not reach the endpoint when the last questionnaire was filled out were censored at that time. Median follow-up was 12.43 months for continence, and 18.97 months for EF.

**Results and limitations:**

Patients undergoing seLND have a lower chance of regaining both urinary continence [hazard ratio (HR) 0.59, 95% CI 0.39–0.90, *p* = 0.026] and EF (HR 0.28, 95% CI 0.13–0.57, *p* = 0.009). Age at surgery had a significant influence on both continence and EF in multivariate analysis. Major limitation of the study was that no formal preoperative assessment of continence and potency was done.

**Conclusion:**

Extending the LND template beyond the eLND template may cause at least a significant delay in recovery of urinary continence and leads to less recovery of EF.

## Introduction

As there is increasing evidence that radical prostatectomy (RP) is a valid therapeutic option in patients with prostate cancer (PCa), possibly providing better oncological results than radiotherapy (RT) (1), it is important to define how extensive this surgery should be to achieve maximal oncological control, especially in high-risk PCa patients. High-risk PCa is defined as Gleason score 8–10 or prostate-specific antigen (PSA) >20 ng/ml or stage ≥T2c according to EAU guidelines (2). Yet, a recent systematic review of the literature showed no clear survival impact of lymph node dissection (LND) after RP (3). Nevertheless, emerging evidence suggest that a more extensive LND may improve PCa-specific survival in node-positive patients (4). On the other hand, current evidence suggest to consider very extensive LND only for patients who are in greatest risk to harbor lymph node invasion of PCa (5).

In high-risk and selected intermediate-risk PCa patients, the EAU guidelines recommend performing an extended LND (eLND) including nodes overlying the external iliac artery and vein, the nodes within obturator fossa located cranially and caudally to the obturator nerve, and the nodes medial and lateral to the internal iliac artery (6). Joniau et al. stated in 2013 that although performing eLND would correctly stage 94% of patients, it would remove all positive pelvic lymph nodes in only 76% of patients, thus possibly achieving suboptimal long-term results (7). Therefore a new, super-extended LND (seLND) was advocated, adding dissection of the common iliac and presacral regions to the existing template.

Patients who are treated with RP may experience functional complications, especially urinary incontinence and erectile dysfunction (8). The possible impaired sexual function recovery after eLND has been hypothesized, but no effect on urinary function recovery was seen in recent studies (9, 10). Nodes at the level of the internal iliac vessels and the presacral region are in close proximity to the hypogastric plexus that contains also parasympathetic innervation to small pelvis and therefore dissection of these nodes may impair erectile function (EF) (11, 12).

In this retrospective analysis of prospectively collected data, we aim to determine whether performing a seLND has a significant influence on recovery of urinary continence and EF when compared to an eLND. To our knowledge, there are no previous studies investigating functional outcomes in relation to seLND during RP.

## Patients and Methods

### Patient Population

The patients included in this study are part of a larger database, which is being assembled at the University Hospitals Leuven, starting in January 2007, including patients who underwent surgery for high-risk PCa, or in whom at least an eLND was performed. The study population consisted of patients included to two different prospective studies (13, 14) (seLND) and from patients treated according to current clinical guidelines (eLND). All surgeries were open RP’s performed by two experienced surgeon (SJ and HP). From January 2007 until December 2013, 526 patients matched the criteria. The patients were followed 3 monthly for the first year after the surgery and then semiannually during the years 2–5 and annually thereafter. During every routine follow-up visit, all patients were handed standard questionnaires (Data Sheet S1 in Supplementary Material) assessing functional outcome. All 172 patients who filled out two or more questionnaires were included in this analysis. Every questionnaire was reviewed and results were registered. Two out of 172 patients were excluded from EF analysis because they did not answer the questions concerning EF. Primary endpoints were postoperative continence and EF. Continence was defined as no loss of urine loss of urine during the day or during the night and no use of any pads. EF was defined as the ability to have an erection sufficient for successful vaginal intercourse with or without PDE-5 inhibitors. Patients who did not reach the primary endpoint at the time the last questionnaire was filled out were censored at that time. Study variables that were analyzed in order to assess their predictive value for continence and EF were eLND, seLND, nerve sparing (NS) surgery, adjuvant radiotherapy (aRT), and age at surgery. The study protocol was approved by the IRB of the University Hospitals Leuven.

### Statistical Analysis

Inverse Kaplan–Meier survival analysis was used to show functional recovery over time. Log-rank test was used to establish whether there was a significant difference between both groups (*p*-value < 0.05). We analyzed the results at three different time points: after 6 months, to establish early recovery, and at 18 and 30 months after RP. We also performed Kaplan–Meier analyses to assess continence and EF recovery over time for in different groups of patients: including NS versus no nerve sparing (NNS) surgery (in this analysis, NS surgery was defined when at least one neurovascular bundle was spared after surgery) and aRT (for practical reasons, this was defined as RT received before the endpoint was reached). There were a significant proportion of men with preoperative ED both in eLND and seLND group and therefore we did not exclude these patients from further analysis based on preoperative ED. Then, we used a Cox proportional-hazards regression to predict which variable could influence, in a multivariable model, continence and EF recovery. All factors shown to have a significant influence on functional recovery were then included with one exception: aRT in EF recovery, since it was possibly associated with androgen deprivation therapy (ADT). Age at surgery was also included to multivariate analysis as a known major factor for functional outcome after RP (15). We also performed sensitivity analysis of EF between eLND and seLND on the subgroups of patients with good preoperative EF and with NS technique. We also tested the same variance between aRT patients and patients who did not receive adjuvant treatments. All statistical analyses were performed using Medcalc software version 12.5.0 (MedCalc Software bvba, Ostend, Belgium).

## Results

### Patient Data

**Figure 1:**
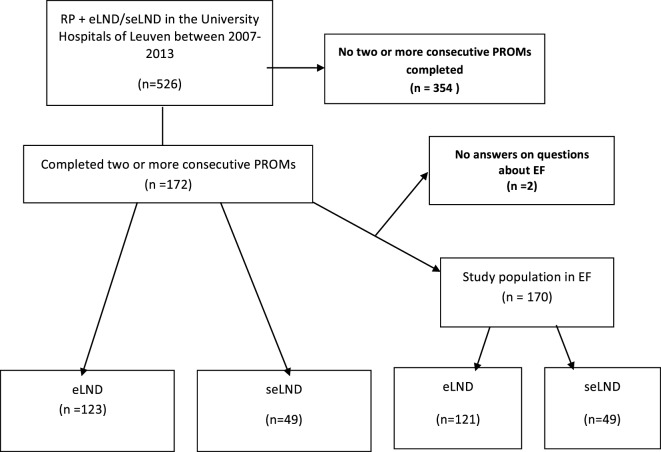
Flow diagram of the study population. RP, radical prostatectomy; eLND, extended lymph node dissection; seLND, super-extended lymph node dissection; EF, erectile function; PROM, patient reported outcome.

The selection criteria for the study are described in the study flow diagram (Figure 1). Of the study population (*N* = 172): 7 (4.1%) had low-risk disease; 51 (29.7%) intermediate-risk disease, and 114 (66.3%) high-risk disease. Median PSA prior the surgery was 8.5 ng/mL (0.9–71.4 ng/mL); median age of the population was 63.1 years (46.8–76.2 years). A NS procedure was performed in 114 (66.3%): 90 (73.2%) in the eLND group and 24 (49.0%) in the seLND group, due to the more advanced tumor characteristics in this population. The median number of removed lymph nodes was significantly higher in the seLND versus eLND group: 24 [interquartile range (IQR) 22–27] versus 15 (IQR 13–17); *p* < 0.001, respectively. By the same definition, we used to define EF after surgery, 52/170 patients (30.6%) reported suffering from some degree of ED even before surgery (Table 1).

**Table 1:** Patient and disease characteristics of the study population.

Characteristics	All patients (*n* = 172)	eLND (*n* = 123)	seLND (*n* = 49)
Age, years, median (range)	63.11 (46.76–76.15)	61.92 (46.76–76.15)	66.89 (49.19–73.84)
Preoperative PSA, ng/mL, median (range)	8.45 (0.94–75.58)	7.25 (0.94–71.44)	11.14 (1.49–75.58)
Clinical T stage (%)			
≤2a	61 (35.1)	54 (43.9)	6 (12.3)
2b–2c	26 (15.2)	21 (17.1)	5 (10.2)
3a	65 (38.0)	37 (30.1)	29 (59.2)
3b–4	20 (11.7)	11 (8.9)	9 (18.3)
Biopsy Gleason Score (%)			
≤6	23 (13.4)	21 (17.1)	2 (4.0)
7 (3 + 4)	63 (36.6)	47 (38.2)	16 (32.7)
7 (4 + 3)	40 (23.3)	27 (21.9)	13 (26.5)
≥8	46 (26.7)	28 (22.8)	18 (36.6)
Risk stratification, EAU (%)			
Low	7 (4.10)	7 (5.7)	0 (0.0)
Intermediate	51 (29.7)	47 (38.2)	4 (8.2)
High	114 (66.3)	69 (56.1)	45 (91.8)
Nerve sparing surgery (%)	114 (66.3)	90 (73.2)	24 (49.0)
Preoperatively described ED, *n* = 170^a^ (%)	52(30.6)^a^	29(23.6)^a^	23(46.9)^a^
Number of lymph nodes removed according to template	eLND	seLND	
Median (IQR)	15 (13–17)	24 (22–27)	*p* < 0.001

*^a^Nominal values from erectile function study population*.

*PSA, prostate-specific antigen; ED, erectile dysfunction; IQR, interquartile range; eLND, extended lymph node dissection; seLND, super-extended lymph node dissection; EAU, European Association of Urology*.

### Urinary Continence

**Figure 2:**
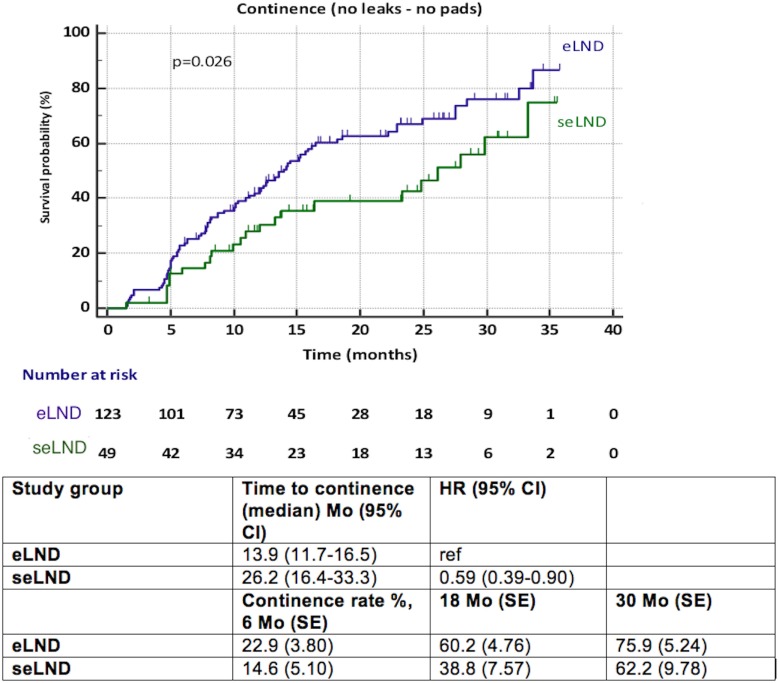
Recovery of urinary continence after radical prostatectomy among patients with extended lymph node dissection (eLND) versus super-extended lymph dissection (seLND) during surgery. HR, hazard ratio, Mo, months, CI, confidence interval, ref, reference, SE, standard error.

Patients submitted to seLND [hazard ratio (HR) 0.59, 95% CI 0.39–0.90, *p* = 0.026] showed statistically significant delay in full continence recovery compared to those submitted to eLND, increasing the median time to recovery of continence from 13.9 months (95% CI 11.7–16.5) in the eLND group to 26.2 months (95% CI 16.4–33.3) in the seLND group. However, at 30 months after surgery, there was decrease in variance between both groups, with 75.9% full continence rate in the eLND group (SE 5.30%), and 62.2% in the seLND group (SE 9.78%). Nonetheless, this was also due to low number of patients at risk at this point (Figure 2).

Neither NS technique nor aRT showed to be significantly related with delayed continence recovery (Figures S1 and S2 in Supplementary Material).

#### Multivariate Analysis

Multivariate analysis showed only seLND and age at the surgery to be significant negative predictors of full continence recovery, both *p* < 0.03 (Table 2). Age at surgery had the most important influence on continence recovery. The chances of recovery of continence seem to diminish with age by 3.6% every year (HR 1.04, 95% CI 1.01–1.07, *p* = 0.016).

**Table 2:** Multivariate analyses for continence and erectile function using Cox proportional-hazards regression.

	Covariate	*p*-value	Hazard ratio (HR)	95% CI of HR
Continence	seLND	0.027	0.579	0.356–0.939
	Age at surgery (years)	0.016	1.036	1.007–1.067
	aRT	0.271	0.726	0.410–1.285
	NS	0.830	0.949	0.590–1.526

Erectile function	seLND	0.019	0.280	0.097–0.812
	Age at surgery (years)	0.005	1.079	1.023–1.139
	NS	0.204	1.895	0.706–5.082

*seLND, super-extended lymph node dissection; aRT, adjuvant radiotherapy; NS, nerve sparing; CI, confidence interval*.

### Erectile Function

**Figure 3:**
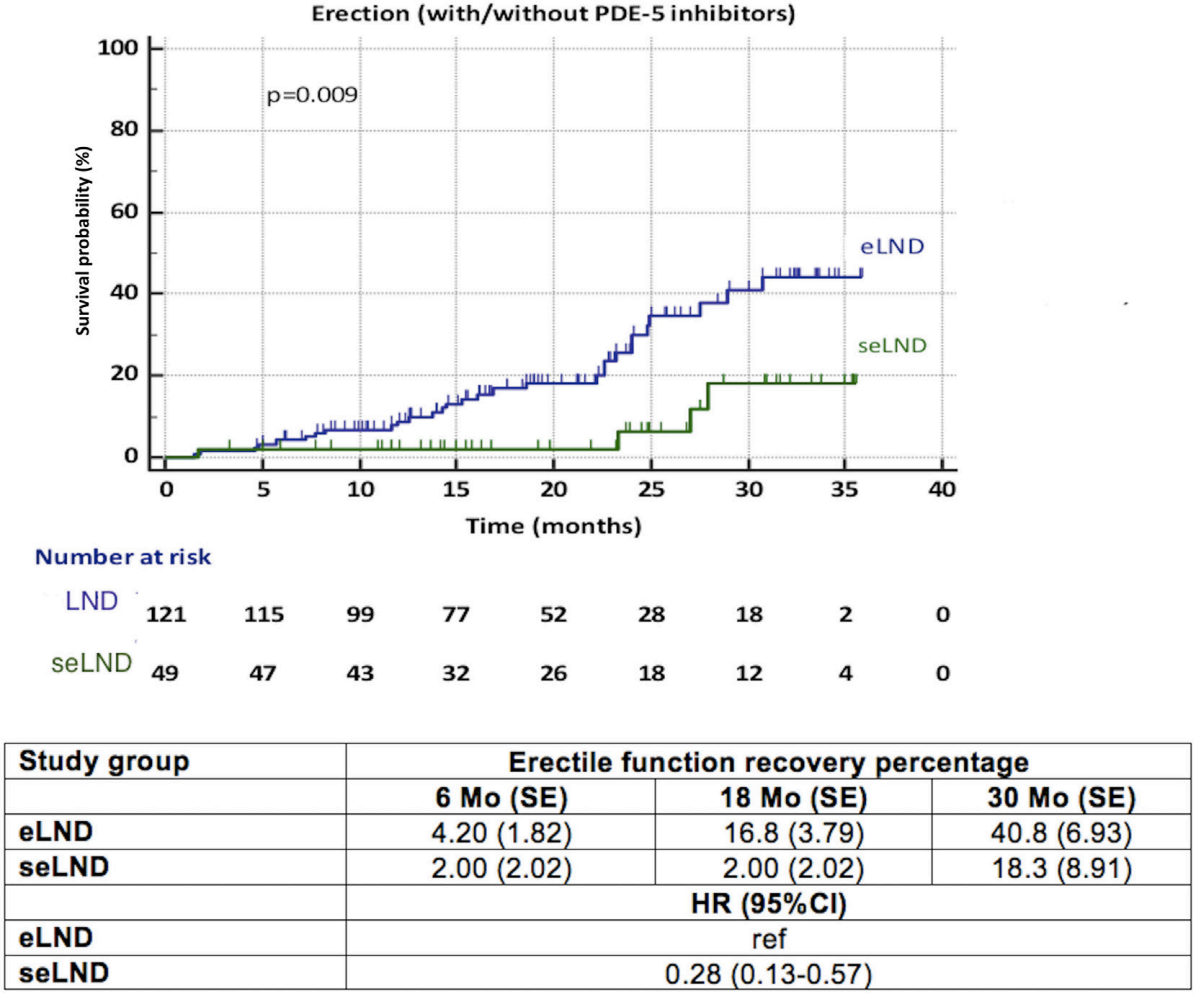
Recovery of erectile function after radical prostatectomy among patients with extended lymph node dissection (eLND) versus super-extended lymph node dissection (seLND) during surgery. HR, hazard ratio, Mo, months, CI, confidence interval, ref, reference, SE, standard error.

When comparing the seLND and eLND group (Figure 3), a significant difference was observed (HR 0.28, 95% CI 0.13–0.57, *p* = 0.009). The median time to EF recovery could not be calculated from our data, since less than 50% of patients reached the endpoint in either group. At 6 months follow-up, 4.2% (SE 1.83%) of patients in the eLND group were capable of having erections sufficient for intercourse, compared to 2.0% (SE 2.02%) of patients in the seLND group. At 18 and 30 months after surgery, 16.8% (SE 3.79%) and 40.8% (SE 6.93%) of patients in the eLND group reached the endpoint, respectively. Respective numbers in the seLND group were 2.0% (SE 2.02%) and 18.3% (SE 8.91%) (Figure 3).

**Figure 4:**
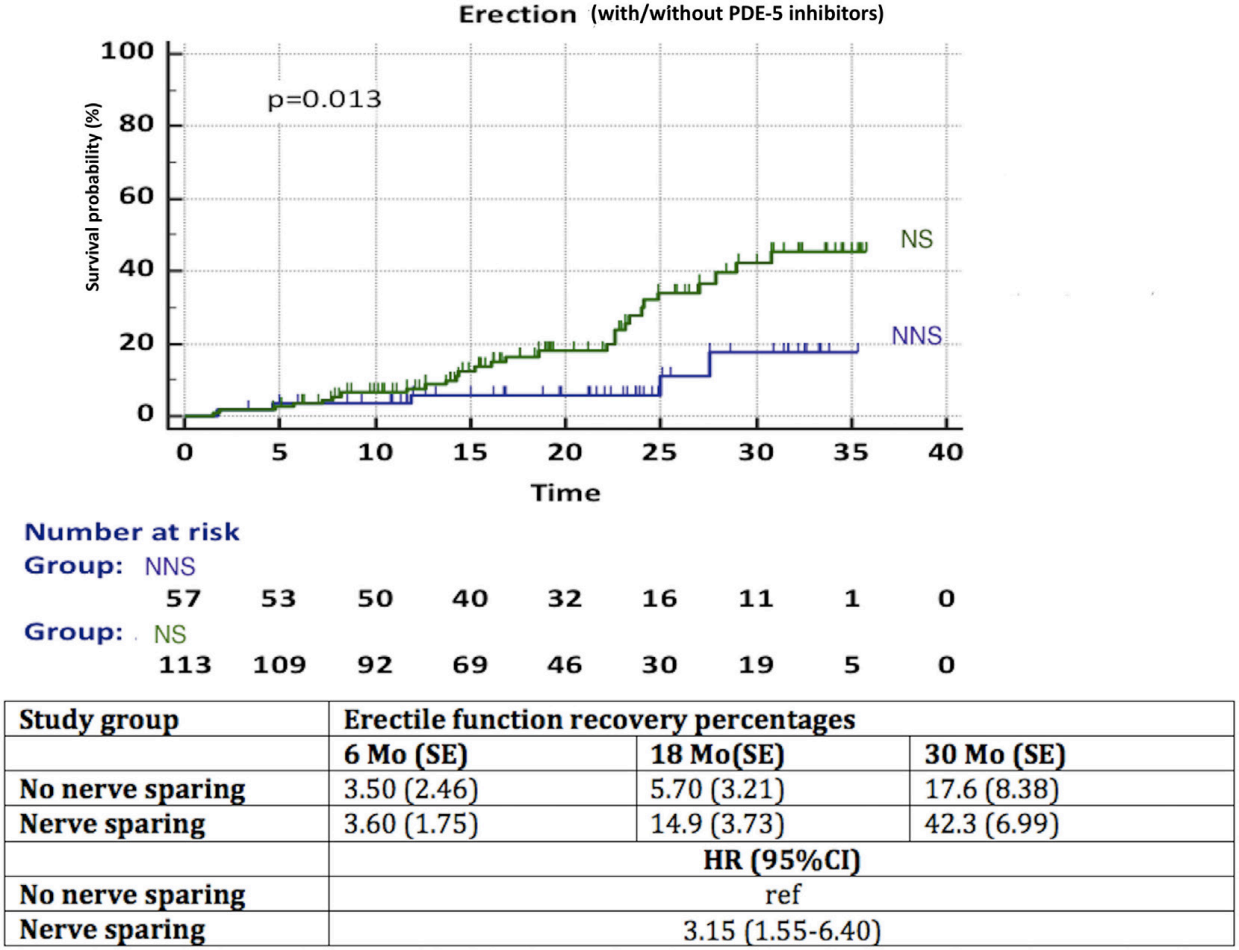
Recovery of erectile function in time among patients with nerve sparing (NS) versus no nerve sparing (NNS) technique during radical prostatectomy. HR, hazard ratio, mo, months, CI, confidence interval, ref, reference, SE, standard error.

**Figure 5:**
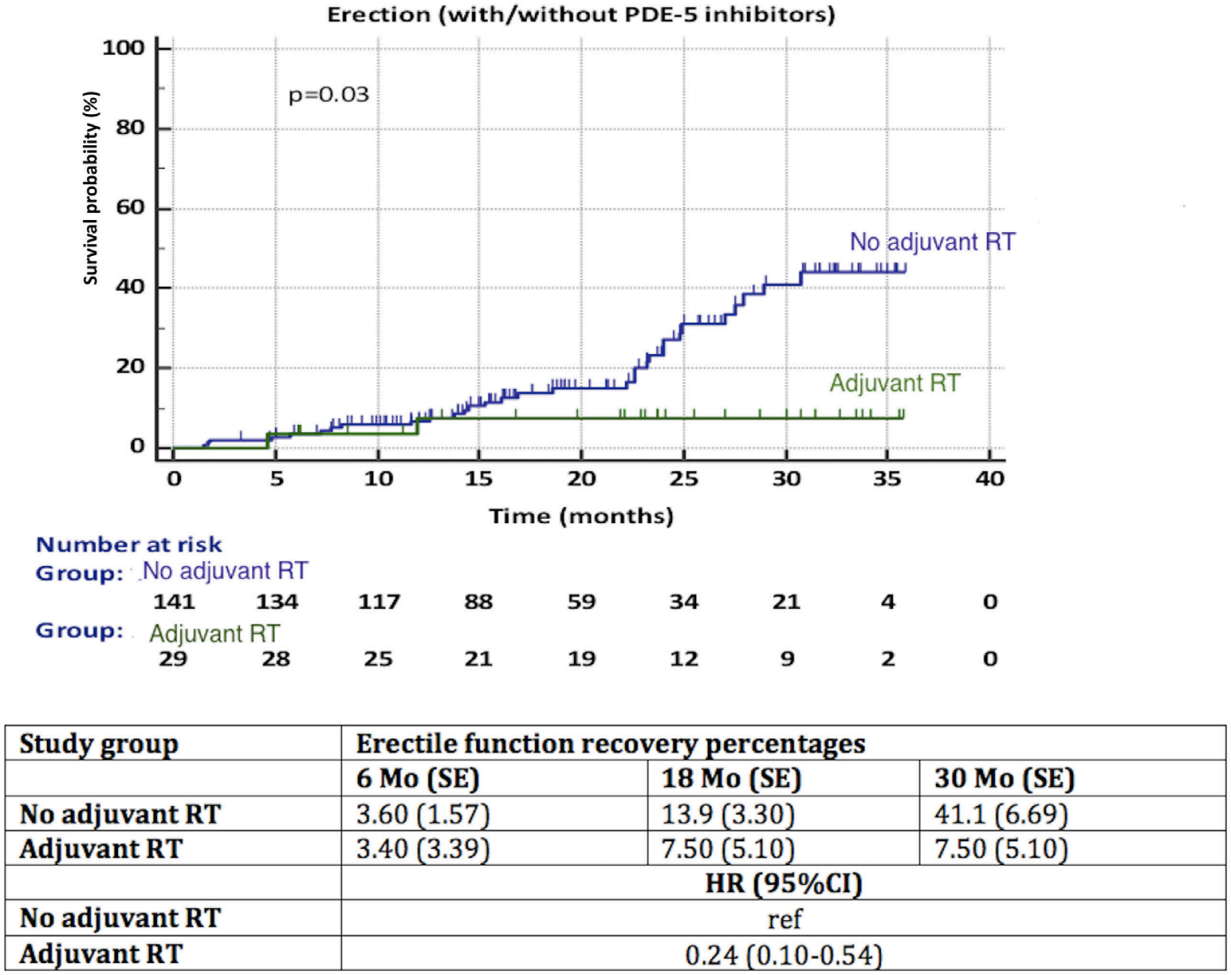
Recovery of erectile function among patients with adjuvant radiotherapy (aRT) versus no aRT. HR, hazard ratio, mo, months, CI, confidence interval, ref, reference, SE, standard error.

A significantly better recovery of EF was observed in patients with NS compared to NNS (HR 3.15, 95% CI 1.55–6.40, *p* = 0.013) (Figure 4). Again, less than 50% of patients in each group reached the endpoint, thus the median time to recovery of EF could not be calculated. We observed a significant influence (*p* = 0.03) of aRT on recovery of EF (Figure 5). It is difficult to draw conclusions from this, as patients who underwent aRT possibly had recurrent disease and may also have received ADT.

#### Sensitivity Analysis of EF

**Figure 6:**
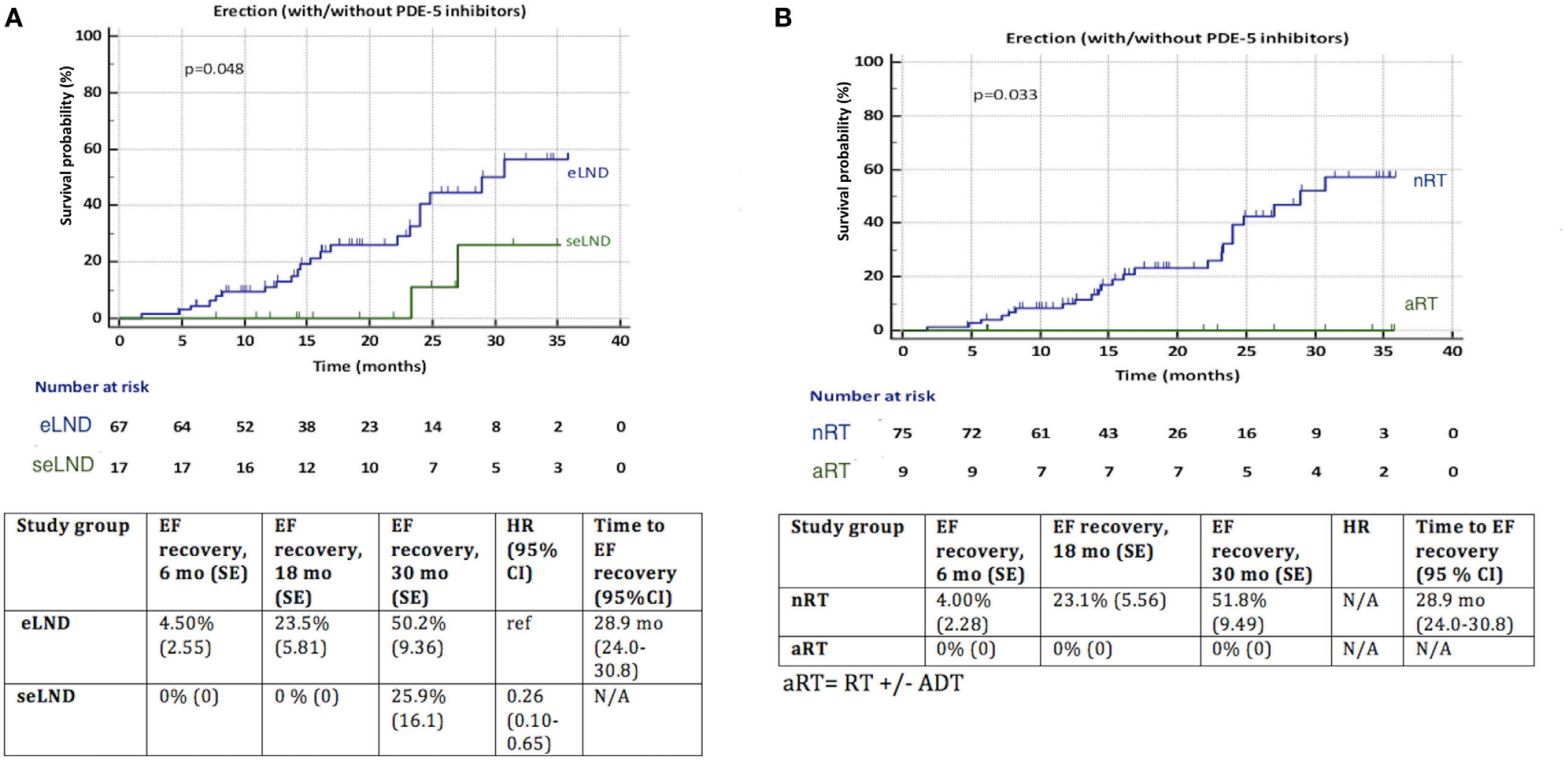
**(A)** Recovery of erectile function (EF) after radical prostatectomy (RP) among patients with extended lymph node dissection (eLND) versus super-extended lymph node dissection (seLND) during surgery. Sensitivity analysis: only patients with good preoperative EF and nerve sparing (NS) surgery. **(B)** Recovery of EF after RP among patients with adjuvant radiotherapy (aRT) ± androgen deprivation therapy versus no radiotherapy (nRT). Sensitivity analysis: only patients with good preoperative EF and NS surgery. HR, hazard ratio, mo, months, CI, confidence interval, ref, reference, SE, standard error.

Patients with eLND and NS during RP with good preoperative EF regained their EF significantly better (*p* = 0.048) compared to the seLND group (Figure 6A). Among patients without aRT with NS and good preoperative potency, EF recovered 51.8% of cases compared 0% in the aRT group, *p* = 0.033 (Figure 6B).

#### Multivariate Analysis

At multivariate analysis, seLND and age at surgery (both *p* < 0.002) were found to be independent negative predictors of EF recovery. Conversely, NS was not an independent predictor of postoperative EF recovery (*p* = 0.20) (Table 2).

## Discussion

To our knowledge, this is the first analysis dealing with functional outcomes after performing a seLND during RP. We observed a delay in continence recovery in patients submitted to seLND compared to eLND. Similarly, there was a striking influence of seLND on EF recovery, with chances of EF sufficient for vaginal intercourse diminishing by about 70% compared to the eLND group. Previous research has demonstrated a benefit of seLND in terms of tumor staging (7) and possibly even PCa-specific survival in high-risk PCa patients (4).

The observation that seLND significantly impaired continence recovery might possibly be explained by an increased damage of nerve fibers innervating the pelvic floor muscles. One recent retrospective study showed that eLND compared to limited LND was not associated with an increased risk of postoperative erectile dysfunction, risk of incontinence or time to continence recovery (16). van der Poel et al. demonstrated that eLND was associated with impaired postoperative EF recovery but not with continence recovery after robot-assisted RP (9). Their findings were based on 1-year of follow-up. Importantly, these studies only assessed possible negative effects of eLND, while seLND was not assessed.

Even though in our analysis aRT showed significant impact only for EF, the use of aRT has a known adverse effect in both major functional outcomes (17–19). Recently, Suardi et al. reported substantially lower continence rates 30 months after RP in patients with aRT compared to patients without aRT (20). Furthermore, a recent retrospective study stated that patients who received aRT had a 4% higher overall incontinence rate 3 years after surgery compared to matched RP-only patients. Moreover, ADT further increased overall and severe incontinence (21). Conversely, former prospective study found no association between increased incontinence rates and aRT (22). We also observed that older age at surgery was a significant predictor for delayed continence recovery. Indeed, the effect of increasing age and reduced continence outcome after RP is well described (15, 23–25). Whereas the influence of RT on continence rates is probably due to a direct toxic effect, the effect of age on recovery of continence is most likely multifactorial, including increased muscle weakness, decreased mobility, cognitive function, etc. Of note, the prevalence of urinary leakage in the general population is more common in elderly men (26). While in our analysis NS showed only to have a significant effect on EF, it has also been proposed to have a role in continence recovery after RP (27). Kaye et al. found a positive effect on continence when performing at least unilateral NS after laparoscopic or robot-assisted RP (28). However, others reported that in open RP, this difference was not observed (29). Further, Michl et al. stated that the NS technique was associated with a more meticulous apical dissection, rather than preservation of the neurovascular bundles. This by itself may explain the positive impact of NS on long-term continence rates (30).

Recovery of EF was significantly impaired in the seLND group versus the eLND group. This can partially be explained by the fact that in the seLND group, only 49% of patients were operated in a NS fashion, as compared to 73% in the eLND group. However, even at multivariate analysis, seLND remained an individual predictor of EF. As we stated earlier, the dissecting the lymph nodes in the presacral area which are in close proximity to the inferior hypogastric plexus might be the major factor explaining this observation. Indeed, when exploring the extent of the LND template, there have been studies showing a decrease in postoperative EF recovery when extending the LND template (9). Conversely, Gandaglia et al. reported that as long as bilateral NS was performed, the extent of LND did not influence potency outcomes (31). NS technique at surgery was a significant factor for EF recovery in our study, even though most patients only received a unilateral NS due to the high proportion of high-risk PCa cases. In a previous study of 1,620 patients who underwent open RP, a significant positive impact of NS surgery on EF was observed, and for each age category, patients who received bilateral NS surgery achieved better results than those who underwent unilateral NS. Potency rates ranged from 86% in the youngest age group (<49 years) to 37% in older patients (>70 years) (32). As expected, NS surgery appears to have an important effect on long-term recovery of EF. Moreover, recovery of EF remains possible even more than 2 years after RP (33). Our results indicate that the possibility to regain sustainable erections after RP is diminishing by 8% for each year of age. Others have reported similar results (32, 34).

Despite the fact that our study is the first to highlight the role of seLND in predicting functional outcomes after RP, the study is not devoid of limitations. First, the retrospective nature of the study represents a weakness. Second, no formal preoperative assessment of continence or EF was done. Third, questionnaires were freely handed out at every follow-up consultation, introducing a selection bias, with possibly more motivated or healthier patients filling out more questionnaires and thus being over-represented in this analysis. This selection bias should however not influence the difference between both groups, but rather could change the overall results. Fourth, the study lacks the use of validated questionnaires.

However, it is of note that this study provides to the best of our knowledge the first available data regarding the predictive role of seLND in terms of functional outcomes after RP. Moreover, this study was based on patient reported postoperative functional outcomes providing real-life estimations of continence and EF, thus providing additional strength to our results. Prospective, randomized studies are needed to better define the role of seLND on functional outcome after RP.

## Conclusion

Although seLND has been demonstrated to provide more accurate staging and optimal removal of potentially affected pelvic lymph nodes, we observed that extending LND beyond the eLND template may significantly influence postoperative functional outcomes. However, these findings are retrieved from retrospective cohort with a slightly different patient material between the groups. Thus, the prognostic value of these findings warrants further investigation, but at this point these findings could be useful for counseling patients with high-risk PCa for whom seLND is considered.

## Author Contributions

Conception and design: SJ. Acquisition of data: HS, PJ, and MT. Analysis and interpretation of data: HS, SJ, PJ, MT, and LT. Drafting of the manuscript: HS, PJ, MT, and SJ. Critical revision of the manuscript for important intellectual content: MT, LT, LM, AB, TB, GM, MA, WE, HP, and SJ. Statistical analysis: HS, PJ, and SJ. Obtaining funding: SJ. Supervision: SJ, HP, MA, and WE.

## Conflict of Interest Statement

The authors declare that the research was conducted in the absence of any commercial or financial relationships that could be construed as a potential conflict of interest.

## Abbreviations

(e/se)LND, lymph node dissection (e = extended, se = super extended); RP, radical prostatectomy; EF, erectile function; PSA, prostate-specific antigen; (N)NS, (no-)nerve sparing; aRT, adjuvant radiotherapy; ADT, androgen deprivation therapy.
