# Supplementary material for: Comparison of Functional Outcome after Extended versus Super-Extended Pelvic Lymph Node Dissection during Radical Prostatectomy in High-Risk Localized Prostate Cancer
Source: Front Oncol. 2017 Nov 22;7:280. doi: 10.3389/fonc.2017.00280 (PMC5702642; doi:10.3389/fonc.2017.00280)
Supplement: Supplementary file 1 [file data_sheet_1.docx]

APPENDIX A: Continence questionnaire. (Endpoints used for this analysis are marked in yellow)

During the day:

1a. Do you involuntarily lose urine without apparent reason and without having the urge to void *during the day*?

1. Never
2. Rarely (not more than once a day)
3. Often (more than once a day)
4. Very often

1b. Do you wear protective pads in your underwear *during the day*?

1. Never
2. Yes, as a precaution
3. Yes, because I have to
   1. I don’t use more than one pad per day
   2. I use more than one pad per day

At night:

2a. Do you lose urine *while sleeping*?

1. Never
2. Rarely (less than once a week, or once a week)
3. Often (more than once a week)
4. Very often

2b. Do you wear protective pads in your underwear *at night*?

1. Never
2. Yes, as a precaution
3. Yes, because I have to
   1. I don’t use more than one pad per night
   2. I use more than one pad per night

APPENDIX B: Erectile function questionnaire (Endpoints used for this analysis are marked in yellow)

1a. Which description best fits the situation *before* your prostate was surgically removed?

- 1. I could not get an erection
  2. I could get an erection, but my penis was not hard enough for sexual intercourse
  3. I could get an erection, and my penis was (or would be) hard enough for sexual intercourse

1b. Which description best fits your *current* situation?

- 1. I cannot get an erection
  2. I can get an erection, but my penis is not hard enough for sexual intercourse
  3. I can get an erection, and my penis is (or would be) hard enough for sexual intercourse
