# Supplementary figures and images for: Comparison of Functional Outcome after Extended versus Super-Extended Pelvic Lymph Node Dissection during Radical Prostatectomy in High-Risk Localized Prostate Cancer
Source: Front Oncol. 2017 Nov 22;7:280. doi: 10.3389/fonc.2017.00280 (PMC5702642; doi:10.3389/fonc.2017.00280)

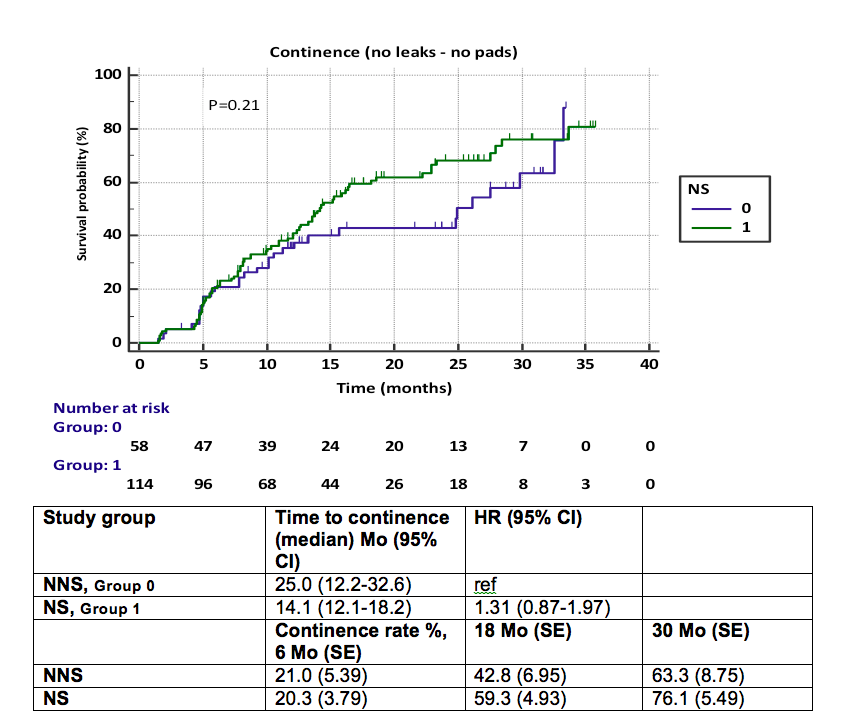

Supplement: Supplementary file 2 [file image_1.tif]

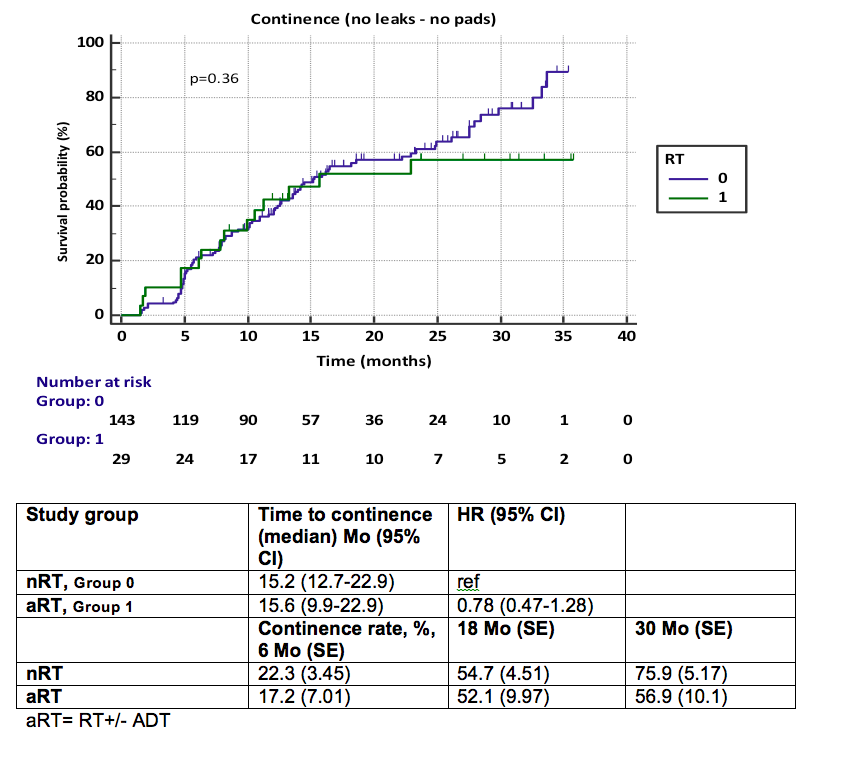

Supplement: Supplementary file 3 [file image_2.tif]
